# Development of Carcinoma of the Cervix Uteri

**DOI:** 10.1038/bjc.1960.18

**Published:** 1960-06

**Authors:** M. M. Boddington, R. H. Cowdell, A. I. Spriggs

## Abstract

**Images:**


BRITISH JOURNAL OF CANCER

VOL. XIV JUNE, 1960 NO. 2

### DEVELOPMENT OF CARCINOMA OF THE CERVIX UTERI

### OBSERVATIONS RESULTING FROM CYTOLOGICAL EXAMINATION OF 10,000 CERVICAL SMEARS

M. M. BODDINGTON, R. H. COWDELL AND A. I. SPRIGGS

*From the Department of Pathology, United Oxford Hospitals.*

Received for publication March 28, 1960

IN 1928 Papanicolaou in New York and Babès in Bucharest independently discovered that carcinoma of the uterus sheds identifiable cells into the vaginal fluid. This finding was later developed by Papanicolaou and others into an important diagnostic method which has now been widely adopted. The cytological diagnosis of carcinoma of the uterus has accumulated an enormous literature, quite out of proportion to the limited information which can be extracted from the examination of vaginal or cervical smears.

It soon became clear that the new method reveals certain abnormalities of the cervical epithelium other than invasive squamous carcinoma. There is, in fact, a whole range of cytological changes corresponding to the histological appearances labelled " atypical hyperplasia " and " carcinoma *in situ* ", and the interpretation of smears immediately became involved in the controversial problem of precancerous change (Ayre, 1952*a*). Since the histological diagnosis is the universally accepted yardstick by which the accuracy of cytological diagnosis is assessed, we have the situation of the blind leading the blind.

The results of experimental work on carcinogenesis show that invasive cancer develops in the course of a series of cellular transformations which are probably discontinuous. After invasion has begun, further transformations may continue to occur, producing increases in malignancy (Foulds, 1958). Among other sites, this discontinuous transformation has been demonstrated experimentally in the cervix uteri of the mouse (von Haam and Scarpelli, 1955 ; Reagan and Wentz, 1959). All the evidence from human material supports the same concept. In one cervix a series of lesions may be found from basal cell hyperplasia or squamous metaplasia up to invasive carcinoma (Howard, Erickson and Stoddard, 1951 ; Gusberg and Moore, 1953 ; Carson and Gall, 1954).

Direct evidence of the precancerous nature of " carcinoma *in situ* " in the human cervix is of two kinds. In the *retrospective* study of Galvin, Jones and Te Linde (1952), out of 13 patients with carcinoma of the cervix who had undergone biopsy one to seventeen years before, a review of the old material revealed " carcinoma *in situ* " in 11. Many *prospective* studies have shown progression of " carcinoma *in situ* " to invasive carcinoma, often after a lapse of many years (Younge, Hertig and Armstrong, 1949 ; Gusberg and Moore, 1953) ; but this progression has not occurred in every case. Unless regression is explained by total removal of the lesion at biopsy, it seems that we are not necessarily dealing with carcinoma at all but with an epithelium which has climbed some of the steps towards malignancy. The data of Petersen (1955; 1959, personal communication) show the proportion of 127 untreated cases which progressed eventually to invasive carcinoma. The cumulative percentage of cases in which carcinoma had developed increased with each year that passed, at first rapidly, then more slowly, and after 7–8 years the risk had become quite small. By extrapolation of the curve it can be deduced that about 35 per cent of cases of " cervical precancerosis " (diagnosed by Petersen) will sooner or later develop carcinoma of the cervix. " Regression " usually occurred in the first year, and 50 per cent of those with lesions persisting beyond this time developed carcinoma within ten years. Whether early invasive carcinoma was present, but not found, at the time of the first biopsy in some of the cases, it is impossible to decide. We also do not know whether the epithelium which developed into carcinoma looked the same as the piece removed at biopsy. These considerations do not invalidate Petersen's figures from the point of view of clinical significance.

If we accept that in only one-third of the cases will "carcinoma *in situ*" progress to invasion, it is important to find out whether the cases can be sorted beforehand into morphological groups carrying different prognoses. The patients are usually young, and many wish for children. Also, if they are all treated as for carcinoma, some may be left with serious complications of treatment (Latour, Brown and Turnbull, 1957). With this in mind we decided to re-examine our material and correlate as far as possible the cytological and histological appearances with the clinical course.

We have also reviewed all the slides from patients whose cervical lesions have been left untreated for a year or more. It seems very important to know whether the abnormal cell-type usually remains the same over long periods, or whether it alters to a more anaplastic type ; and if this happens, whether it happens suddenly or gradually. Data on this point are extremely scarce (Ayre, 1952*a*, *b*) and our series provides evidence about the stability of the cell-type in ten cases.

### PRESENT INVESTIGATION

Between 1952 and 1959 10,000 cervical smears were examined from 8522 cases, most of which were gynaecological outpatients. The smears were taken by the examining gynaecologist by scraping the entire squamo-columnar junction area with a wooden spatula as described by Ayre (1952*a*), and smearing the material on a slide, and one slide was made on each occasion. The slides were fixed in ether-alcohol or 3 per cent acetic acid in ethyl alcohol, and they were stained by the Papanicolaou method.

The histological material available has usually been a ring or multiple biopsy of the squamo-columnar region of the cervix. Sometimes a cone biopsy, the amputated cervix or the whole uterus has been submitted. Whenever possible all the cervical tissue available has been embedded and either serial sections or multiple step sections examined.

During the period of 7 years, 11 cases of clinically unsuspected invasive carcinoma of the cervix were detected, as well as 67 cases with possibly precancerous cervical abnormalities. Of the latter group, 56 were clinically unsuspected.

In the same period there were 9 adequate smears which were " false negative " for cervical carcinoma, and 14 " false positive " in which histological studies revealed no cause for the abnormal cells. In some of these cases, it is possible that the area from which the abnormal cells were derived has not yet been removed for examination.

By reviewing the cytological and histological slides from all these cases we hoped to answer the following questions :

1. Can the histological findings be predicted from the cytological appearances?
2. If invasive carcinoma develops from any of the other lesions, can this be predicted either histologically or cytologically? Which types, if any, regress and which persist unchanged?
3. Is the cytological type consistent over a period of time, or does it change either gradually or suddenly?

### CLASSIFICATION AND TERMINOLOGY

A system of numerical histological and cytological grading was devised in order to avoid any built-in opinion about the significance of the finding or its prognosis. All the histological slides were reviewed and graded by the same observer (R. H. C.) without knowledge of the clinical or cytological findings. The cytological preparations were independently reviewed and graded by two observers (M. M. B. and A. I. S.), and for this purpose the identification marks of the slides were covered.

### A. *Histological Classification*

For the purpose of this investigation we have reviewed all the relevant slides and classified them according to the following numerical system.

*Non-invasive lesions*

1. Simple basal cell hyperplasia, occurring beneath either squamous or endocervical columnar epithelium (Fig. 1).
2. A thick stratified squamous epithelium with hyperkeratosis. Many of the superficial squames are nucleated and there is variation in size of these nuclei, some being quite large, but the general epithelial pattern is orderly and mitoses are few. The distinction from simple hyperkeratosis is based on the somewhat verrucous variation in the width of the epithelium and the variable size of nuclei in keratinised cells (Fig. 2).
3. Similar to 2 with equally well-defined stratification but more mitotic activity and nuclear irregularity (Fig. 3).
4. Epithelial activity at level of 3 but without the marked hyperkeratosis (Fig. 4).
5. Stratified squamous epithelium, often with considerable variation from normal in the thickness of the strata but with all represented. Mitotic activity greater then 4 and moderate numbers of cells in all layers with large hyperchromatic nuclei (Fig. 5).
6. Epithelium showing some stratification, but poorly developed compared with a normal squamous epithelium. Mitoses and abnormal cells numerous. A thin keratinised layer present on the surface (Fig. 6).

7. Stratification virtually absent but still slight superficial keratinisation in some cases. Many mitoses and abnormal cells (Fig. 7).

*Invasive carcinoma*

8. Squamous carcinoma of any grade of differentiation in which the tumour cells tend to lie in irregularly shaped solid aggregates with increasing maturity towards the centre, where keratinisation may be present (Fig. 8).

9. Squamous carcinoma including sheets of strikingly uniform large polygonal cells, similar to prickle cells although usually without intercellular bridges (Fig. 9).

10. Squamous carcinoma with a high proportion of grossly abnormal large cells including giant cells with massive irregular nuclei and numerous mitoses, many of them atypical. These tumours present an appearance similar to that seen in a squamous carcinoma soon after irradiation, but the biopsies given this classification were taken before radiotherapy (Fig. 10).

11. A " transitional " type of tumour with broad bands of cells rather than clumps. The general appearance is of fairly uniform hyperchromatism with no keratinisation, and the individual bands of tumour cells are somewhat reminiscent of the epithelium of a " carcinoma *in situ* " (Fig. 11).

Some explanation is required for the choice of the histological classification. As regards the non-invasive lesions, so many names have been applied by different authors that is has become the fashion to quote a sample of them, discard them and start afresh. Hinselmann (1953), in explaining the reason for formulating his rubrics (Hinselmann, 1928), said that in his grading he consciously avoided controversial terms such as pre-cancerosis and pre-invasive carcinoma, only aiming at a more accurate definition of the existing epithelial atypism. Our motives are similar to his, and the present numerical classification was worked out as an experiment in the course of reviewing the present series. The intention was to separate different histological pictures which might give rise to the different cell types found in smears.

Several authors divide epithelial dysplasia of the cervix uteri into two categories, basal cell hyperplasia and carcinoma *in situ*. In basal cell hyperplasia, a variable proportion of the width of the epithelium consists of basal cells and the severity of the lesion is defined in terms of this proportion (Nesbitt, 1955). We have seen very few examples of this straightforward proliferation. Those found are collected as grade 1.

The verbal criteria for diagnosis of "carcinoma *in situ*" are much better defined than the lesions themselves, and it is frequently reiterated that loss of normal stratification is a constant and essential change (Galvin and Te Linde, 1949). In our opinion, much depends on the word " normal ". Bowen's disease of the skin has a range from complete disorganisation of the epidermal pattern to the presence of abnormal cells scattered in all layers in an otherwise fairly well-organised epithelium. Equally, in the cell nests of an invasive carcinoma there is often stratification from cells of basal type at the margin to keratinised squames at the centre. The present classification indicates *inter alia* the degree to which stratification is maintained and this, like the extent of cellular pleomorphism and mitotic activity, appears to vary in a very gradual manner with no sudden steps from one grade to the next. This is also widely recognised (Foulds, 1958). It is a common custom to describe and illustrate one's criteria with great care, the illustrations covering a single microscopic field, while in practice a diagnosis is reached by examination of the whole of the material available. As regards illustration, the same criticism is inevitable in this series, and it is recognised that the changes seen may vary widely in the same specimen. In general, but not necessarily in detail, the changes in the non-invasive lesions are towards progressively greater " anaplasia " with increasing number, and the number given in each case is the highest applicable. We believe that all workers in this field would regard our grades 6 and 7 as " carcinoma *in situ* ", that many would include grade 5, and that a few would also include grade 4.

The grouping of invasive carcinomata used here has nothing to do with degree of anaplasia as such, but with predominant cell type and arrangement which it was thought might give rise to variations in cell pattern in cervical smears. Grade 8 includes tumours of Broder's Grade 1–4, provided that they conform to the criteria mentioned. The tumours in grade 11 have a pattern much closer to that of " carcinoma *in situ* " than to typical squamous carcinoma.

### B. *Cytological Classification*

The predominant abnormal cells seen in smears were, on review, given a numerical claasification. This has been done only for the purpose of the present investigation and, like the histological grading, is not intended as a new terminology.

0. Insufficient evidence for suspicion of malignancy.

A–F. In all these grades cells were seen with nuclei showing hyperchromasia and some or all of the other aberrations which are found in carcinoma (namely enlargement, multiplicity, folding or wrinkling of nuclear membrane, prominent nucleoli, aggregation of chromatin into numerous evenly or irregularly distributed granules (chromocentres)).

A. Cells have profuse cytoplasm and are polygonal in outline (Fig. 12).

B. Rounded cells with regular cytoplasm and distinct cell borders. Few small forms with high nucleo-cytoplasmic ratio. (Bi- and multinucleation is common in this type) (Fig. 13).

C. As B, but with predominance of cells showing high nucleo-cytoplasmic ratio and/or small irregularly shaped forms (Fig. 14).

D. Nuclei very variable in shape and size, sometimes pyknotic. Cytoplasm often highly keratinised. Bizarre shapes (tadpoles, fibres) (Fig. 15).

E. Large nuclei, high nucleo-cytoplasmic ratio. Cytoplasm shows little or no keratinisation, stains poorly, and is often indistinctly outlined, torn or lost. Some aggregates may be present (Fig. 16).

F. Nuclei rather uniform and crowded, occurring mainly in aggregates. Separate cells have torn cytoplasm, or are necrotic (Fig. 17).

The cytological grades have been chosen with the idea of making broad morphological distinctions not between isolated cells, but between cell-populations in different smears. Only the abnormal cells under consideration have been graded, and no account has been taken of whether they are many or few, or of the associated epithelial or inflammatory cells.

Our numerical grades are not devised to correspond with any previously published system, but previous authors have used similar categories although with variations in nomenclature.

Papanicolaou (1954) divides the cells desquamating from cervical carcinoma into two main types. Firstly, those of " early malignancy " show nuclear changes but a more or less normal cytoplasm (our grades A, B, C). These " dyskaryotic " cells may be of superficial, navicular or intermediate, parabasal, or endocervical type. Dyskaryosis is shown in cases of intra-epithelial carcinoma or in cases of early invasion, but the superficial and intermediate type may only reflect epithelial dysplasia, and are more likely to regress spontaneously. Secondly, the cells of " advanced malignancy " show cytoplasmic as well as nuclear changes, especially elongation ; they also often occur in large clusters.

Graham and her co-workers (Vincent Memorial Laboratory, 1950) have given a rather different classification, into " undifferentiated cells " whose cytoplasmic borders are indistinct, and " differentiated " malignant cells with distinct cytoplasmic borders. The second group includes " fibre cells " and "tadpole cells " which Papanicolaou would classify as cells of advanced malignancy, and " third type differentiated cells " with a high nucleo-cytoplasmic ratio, which Papanicolaou would include under " dyskaryosis ", and come into our grade C. Graham's classification is followed by Zinser (1957).

For Graham (1957) the distinction between third type differentiated and non-malignant dyskaryotic cells is one of actual measurement, the malignant cells having a nucleus whose maximum diameter is larger than its distance from the cell border.

Ayre (1952) describes a " precancer-cell-complex " which corresponds roughly to Papanicolaou's " dyskaryosis " and our grades A, B and C, although some " dyskaryotic " cells would appear to fall into Ayre's group of well differentiated cancer cells. Cases of intra-epithelial carcinoma either show " precancer cell complex " or " cancer cells of pre-invasive type ".

It has been maintained (Nieburgs and Pund, 1950 ; Scapier, Day and Durfee, 1952 ; Reagan, Hamonic and Wentz, 1957) that a distinction between carcinoma *in situ* and invasive carcinoma can be made from the cytological appearances. Reagan and his collaborators have made numerous measurements of cervical cells and report that invasion is associated with a change from rounded to elongated or caudate forms, and that this feature is particularly noticeable in the keratinising growths induced by carcinogens in the cervix of the mouse (Reagan and Wentz, 1959). The differences between the smear patterns of carcinoma *in situ* and invasive cancer have been codified by Wied (1956), but the presence or absence of blood, pus and Döderlein's bacilli are considered as well as purely cytological characters, and there must be few who feel any great confidence in making such a forecast. Mackenzie (1955) analysing the cytological appearance in 27 cases of carcinoma *in situ*, found in 15 a predominance of Graham's " third type differentiated " cells, in 9 a picture of bizarre highly differentiated cells exactly as is seen in advanced cancer, and in 3 marked " dyskaryosis " without any cells considered malignant.

McCormack, Belovich and Krieger (1957) have classified the cells found in invasive carcinoma, and describe three main groups, the first two of which correspond to Martzloff's (1923) terminology. The " spinal-cell " group covers the highly keratinised pleomorphic types ; the transitional (the largest group), shows an admixture of large and small forms ; and the small-cell group corresponds to the most anaplastic histological type. Carcinoma *in situ* could not be distinguished with any certainty from invasive cancer.

CYTO-HISTOLOGICAL CORRELATION

For every observation a histological slide was graded as well as the smear taken shortly before. If the two cytological observers gave different gradings, each was entered as a half. More than one entry by the same observer was similarly fractionated. A separate histogram has been made for each histological grade, showing the distribution of cytological assessments found in that grade.

The numbers in each cytological grade are expressed as percentages of the total number for that histogram, so that every histogram totals 100. (The absolute number involved is given on the right.)

Where several observations were made on the same patient, they are treated independently for this purpose.

Histological grade 0 has been omitted. The cases were deliberately selected for cytological abnormality, and obviously the figures cannot be used to show the distribution of cytological findings in cases with normal cervical histology. It is likely that these were biopsies in which the relevant area was missed.

The following deductions can be made from the histograms :

(1) Most histological grades have a corresponding modal cytological grade, but there is so much variation from this that it can never be justifiable to deduce the histology from smears. Even the most sinister-looking cells can be shed from areas of midly unstable epithelium.

(2) On the average, the histological range from slight instability to anaplastic carcinoma is matched by a cytological range, but some types are more characteristic than others. For instance, cytological grades A and B were characteristic of histological grades 1, 2 and 3, and were very seldom found in cases of invasive carcinoma. On the other hand histological grade 7, without invasion, was associated with a very similar distribution of cytological grades to that shown by the most anaplastic invasive carcinomata (Grade 11). Moreover, well-differentiated squamous carcinomata (Grade 8) were matched on the whole by lower cytological gradings than were the more anaplastic-looking examples of " carcinoma *in situ* " (Grade 7).

It would be a pity to give the impression that the cytological appearances are of no interest beyond indicating the need for a biopsy. They certainly reflect cellular changes which are of biological importance. They do not, however, give any clue to the invasive properties of the deeper parts of the epithelium, of which they represent only the superficial layer.

### OBSERVATIONS ON CASES WITH EPITHELIAL INSTABILITY OF THE CERVIX

Sixty-seven cases were seen in which the cervix showed an abnormality short of invasive carcinoma, but classifiable in the histological grades 1–7 described above. Because we do not know where to draw the line between " carcinoma *in situ* " and lesser abnormalities, they will all be included under the heading of " epithelial instability ".

In three cases an invasive carcinoma was found at least a year after the first smear. One of these (Case 5) is included although there was no histological evidence of epithelial instability, but grade E cells were present in smears taken two years before the carcinoma was discovered.

There were no deaths or known recurrences following treatment.

In the majority of cases where smears were taken after the biopsy, and before any other surgical procedure, the abnormal cells were found to have disappeared. This was observed in 15 cases, and these have continued to have negative smears. This group represents *either* spontaneous regression *or* complete removal of the

---

### EXPLANATION OF PLATES

FIGS. 1–11.—Sections of cervical epithelium representative of the gradings used in this study. (H. and E.)

FIG. 1.—Grade 1. ×110.

FIG. 2.—Grade 2. ×40.

FIG. 3.—Grade 3. ×80.

FIG. 4.—Grade 4. ×110.

FIG. 5.—Grade 5. ×110.

FIG. 6.—Grade 6. ×110.

FIG. 7.—Grade 7. ×110.

FIG. 8.—Grade 8. ×55.

FIG. 9.—Grade 9. ×110.

FIG. 10.—Grade 10. ×55.

FIG. 11.—Grade 11. ×55.

FIGS. 12–17.—Papanicolaou-stained cervical smears showing cells representative of the gradings used in this study. ×350.

FIG. 12.—Grade A.
FIG. 13.—Grade B.
FIG. 14.—Grade C.
FIG. 15.—Grade D.
FIG. 16.—Grade E.
FIG. 17.—Grade F.

FIG. 18.—Case 2, first smear. ×350.

FIG. 19.—Case 2, smear taken three years later. ×350.

FIG. 20.—Case 4, first smear. ×350.

FIG. 21.—Case 4, smear taken a year later. ×350.

FIG. 22.—Case 5, first smear. ×350.

FIG. 23.—Case 5, smear taken two years later. ×350.

FIG. 24.—Case 6, first smear. ×350.

FIG. 25.—Case, 6, smear taken almost three years later. ×350.

FIG. 26.—Case 9, first smear. ×350.

FIG. 27.—Case 9, smear taken six months after the first. ×350.

FIG. 28.—Case 9, smear taken fifteen months after the first. ×350.

1

2

3

4

5

6

Boddington, Cowdell and Spriggs.

7

8

9

10

11

Boddington, Cowdell and Spriggs.

12

13

14

15

16

17

18

19

20

21

22

23

24

25

26

27

28

lesion at biopsy. In three other cases the smears were still positive after the first biopsy, but became negative after subsequent ones.

Usually the course of the disease was interrupted by surgical treatment, and nothing more can be learnt from these cases. There was, however, a group of ten cases in which, for various reasons, a persisting lesion was followed by repeated smears for at least a year. In four of these the period of observation was 2–3 years, and in one was 5 years. These are a selected group, but are of particular interest because the literature contains very few details about the changes, if any, to be found in the desquamated cells over periods of time. Is there a continuous transformation towards more anaplastic types? Or are such transformations sudden, punctuating long periods of morphological stability? Or does the cell-type remain unchanged, even if invasion supervenes? The following brief description accompanied by photographs show the different patterns of cellular behaviour observed in this series.

A. *Persistence of abnormal cells without cytological progression**

Case 1 (Reg. No. 35470) aged 33.—Complaint—irregular excessive bleeding. Six smears were taken over a period of 3½ years. The first four showed cytological grades A + C, but two taken in the last year showed little abnormality. Three biopsies all showed grade 6 lesions. Hysterectomy was finally performed, and sections of the cervix showed areas of grade 6 and of more extensive grades 3 and 1.

Case 2 (Reg. No. 34924) aged 49.—Complaint—irregular periods. The cervix was eroded. The smear showed grade A cells (Fig. 18). She failed to attend for biopsy and was next seen 3 years later. A further smear still showed grade A cells (Fig. 19) and a biopsy showed a grade 3 lesion. She is still being followed.

Case 3 (Reg. No. 29734) aged 38.—Complaint—irregular prolonged periods. Normal looking cervix. Cytological grade C. A biopsy showed a grade 4 lesion. She was not seen for 5 years, after which another smear showed abnormal cells but now of grade B. A recent biopsy shows no abnormality.

Case 4 (Reg. No. 29653) aged 33. Complaint—vaginal discharge. A small erosion was present. Smears graded E–F (Fig. 20), biopsy grade 6. The same cell type was repeatedly found in smears taken over a period of a year (Fig. 21) and a second biopsy 10 months after the first was again grade 6. Hysterectomy was then performed, and no atypical epithelium remained in the specimen.

Case 5 (Reg. No. 33994) aged 35.—First seen in early pregnancy with a complaint of bloodstained discharge. There was a vascular erosion. Smears showed grade E cells (Fig. 22). Biopsy was not done at this time, and two smears taken later in pregnancy were reported as negative, but on review one of them shows similar cells. Two years later similar abnormal cells were still present (Fig. 23), and a biopsy revealed an early carcinoma (grade 8). A Wertheim's hysterectomy was then performed.

Case 6 (Reg. No. 21090) aged 33.—Complaint of vaginal discharge. The cervix was eroded. Smears showed grade B–C cells (Fig. 24), biopsy grade 1. She was followed with repeated cervical smears for nearly three years, during which time she had two pregnancies (one ending in abortion, the other ectopic). The same type of cell was always present, though fluctuating in numbers (Fig. 25). Thirty-

* " Cytological progression " means alteration of the abnormal cell type to a higher grade, whether or not the lesions became invasive.

four months after the first smear, a biopsy showed a very small carcinoma (grade 8).

Case 7 (Reg. No. 32352) aged 37.—Complaint—dyspareunia. Smears showed grade C cells, and biopsy showed a grade 1 cervical epithelium. During the following year two further smears were taken, without appreciable change, and two further biopsies showed no atypical epithelium. Unfortunately no further smears were obtained during the following year, but a hysterectomy was performed two years after the first smear on account of menorrhagia and pelvic pain, and the cervix contained a very small grade 8 carcinoma.

The series of smears is incomplete in this case, but the original cell type persisted for at least a year.

Case 8 (Reg. No. CG/3475) aged 29.—At post-natal examination a large cervical erosion was found, and smears showed grade E–F cells. A biopsy showed a grade 7 lesion. As the smears continued positive for 2 months without change of cell type, the cervix was amputated. The specimen could not be graded owing to previous cauterisation. Subsequent smears still showed abnormal cells, but these were of a different sort (grade B). Another biopsy 6 months after the amputation showed no abnormality, but a third after a further 6 months showed a grade 5 lesion. The cellular picture remained unchanged, and a total hysterectomy was performed, retaining the ovaries.

After this the smears from the vaginal vault have remained abnormal (grade A–B). A biopsy taken from the vault 5 years after the first shows an unstable vaginal epithelium (grade 5).

B. *Progression of cell type during observation*

Case 9 (Reg. No. 33443) aged 28.—Complaint of infertility. Normal cervix. Smear showed cells of grade C (Fig. 26). Biopsy showed a grade 5 lesion. A series of 15 smears were taken over a period of 15 months, during which a normal pregnancy and delivery took place. Six months after the first smear the abnormal cells began to become more crowded, with a reduction in their cytoplasm (Fig. 27). The last smear of the series, taken 3 months post-partum, showed abundant grade F cells (Fig. 28) and cervical amputation was performed. The sections showed a grade 7 lesion.

C. *Regression after persistence for a year*

Case 10 (Reg. No. 29506) aged 27.—Complaint—dysmenorrhoea. There was a third degree tear, and she was admitted for repair. At this time a cervical smear showed grade C–E cells. Repeated smears showed the same type of cells, and a biopsy 8 months after the first smear showed a grade 5 lesion. The abnormal cells persisted for a further 4 months, when a second biopsy was done. This showed only chronic cervicitis, and all subsequent smears were negative during an observation period of 4 years. Two further biopsies showed chronic cervicitis only.

This series of cases illustrates three points.

1. The same cytological type often persists with only slight fluctuations over considerable periods of time, and does not show continuous progression towards more anaplastic types.

2. Invasion can almost certainly supervene in areas of epithelial instability, although it is impossible to prove that the lesion was not invasive at the outset.

In three of the above cases invasive carcinoma was eventually found after at least a year's observation (Cases 5, 6, and 7) and in none of them was any cytological progression observed.

3. In case 9 the abnormal cells showed an unmistakable cytological progression. The smears were not all comparable and it is difficult to be sure whether to record a definite change at 14 weeks pregnancy, but the final smear taken 3 months after delivery showed entirely new appearances, with marked crowding of nuclei and loss of distinct cytoplasmic borders. There was also a histological change from grade 5 to grade 7. This example suggests that morphological alterations, when they occur, proceed stepwise rather than by a continuous transformation. Ayre (1952*b*), in a case studied with over 200 smears, found a " slow progression " until the rather sudden disappearance of the abnormal cells. In his book (Ayre, 1952*a*) changes in cell type are illustrated in " Cell Behaviour Studies " cases 1 and 3, but it is not clear whether the alterations were sudden or gradual ; case 3 showed one distinct change, then long persistence of the same type. Many more cases are required to settle this point.

### DISCUSSION AND CONCLUSIONS

Because of the accessibility of the cervix uteri for smears and biopsies, more is known about the earliest stages leading to cancer at this site than at any other. We are able to recognise lesions which carry a measureable risk (about 30–40 per cent) of sooner or later becoming malignant (Petersen, 1955). As this development probably occurs in a random fashion, it does not seem to be possible to predict the course in any particular case.

Unfortunately, the picture has been confused by disputes about whether this or that histological appearance should be called carcinoma or not. Except when there is evidence of invasion, the question is one of words and not of facts. It is unfortunate that the terms " carcinoma *in situ* ", " intra-epithelial carcinoma " and " stage 0 carcinoma " have been introduced, as they are most difficult to define and no two pathologists will agree about their exact use.

We recommend the term " epithelial instability " to cover all the histological lesions illustrated in Fig. 1–7. Basal-cell hyperplasia (our grade 1) might seem out of place here, but since in two cases (cases 6 and 7) it preceded invasive carcinoma by 2–3 years, we feel that it should be treated with the same respect as the other grades which we have illustrated.

In any case found to have epithelial instability of the cervix it is of the utmost importance to discover whether there is in fact an early invasive carcinoma which has been missed. Several authorities have suggested that a distinction can be made fairly reliably from cytological evidence. We cannot confirm this. Our cytological gradings, while roughly reflecting the cell-types seen in the corresponding histological sections, have not been correlated with the presence or absence of invasion, and we think it wrong to make guesses on the basis of cervical smears. Even the biopsy sometimes fails to reveal an invasive carcinoma ; a full histological study of a ring biopsy of the squamo-columnar junction should be made in every case where this problem arises.

In the present series, the majority of untreated cases ceased to have positive cervical smears after one or more biopsies, and the abnormality has not recurred. It is impossible to determine whether these lesions regressed spontaneously or whether they were completely removed by the biopsy ; most probably there have been examples of each. In ten cases a persistent lesion was followed for over a year, and three of these were eventually found to have small invasive carcinomata. Unfortunately our figures cannot be used to assess the probability of development of carcinoma ; selection was exercised at all stages, and the cases with persistent lesions which remained untreated are only a small residue of the whole series ; also the follow-up period is very short. Our experience has however, been quite consistent with the pattern shown in Petersen's (1955) series ; he found that in approximately 50 per cent of cases with " cervical precancerosis " the lesion disappeared within a year, but that if it persisted, carcinoma developed within 8 years in 50 per cent (13 out of 26).

Are the lesions which regress different histologically or cytologically from those which persist? The analysis of our findings shows no morphological criterion correlated with persistence or regression, nor with the subsequent development of carcinoma. In practice the distinction between persistent and transient lesions is easily made by the use of repeated cervical smears. In the present series those smears which became negative nearly always did so following the first biopsy ; case 10 is the only striking exception.

The series of persistent lesions provides some interesting evidence for the " stepwise " progression of premalignant states. As the paired illustrations show, the abnormal superficial cells shed from areas of epithelial instability are not steadily changing towards a more anaplastic appearance. In the relatively short periods over which we have been able to observe them they have usually remained true to type, just as malignant cells generally do both in experimental conditions and in human disease. In case 9 a sudden change was observed, consistent with the development of a new cell clone. The discovery of invasive carcinoma in two cases while under repeated observation was *not* associated with any alteration in cell type. Evidently, it is unjustifiable to expect any cytological warning before invasion supervenes.

The problem confronting the gynaecologist in these cases is never twice the same, and no generalisations can be made about the appropriate treatment of " epithelial instability ". Above all, no help can be expected from the study of the abnormal smears or biopsies in deciding the prognosis of an individual case. There is no urgency, and provided that the patient is kept under surveillance there does not appear to be any danger. On the other hand, a lesion of this type which does not disappear after biopsy is undoubtedly " precancerous ", in the sense that cancer is sooner or later very likely to develop, and the cervix or the uterus ought to be removed, and negative smears obtained, before the patient is discharged from observation.

### SUMMARY

Now that 10,000 cervical smears have been examined from gynaecological outpatients in Oxford, cytological and histological material pertaining to carcinoma of the cervix and possible precancerous lesions has been reviewed.

The lesions found at biopsy have been given numerical gradings. The different types of abnormal cells seen in the smears have also been classified numerically, and a comparison has been made between the cytological and the histological gradings. Although there is a rough correlation between certain cytological and histological types, the presence or absence of invasion is not correlated with distinct cytological patterns.

Sixty-seven cases were found of possibly precancerous lesions (including " carcinoma *in situ* "). In 33 of these the cervix or uterus was removed surgically without any further observations. In 18 the lesion could no longer be detected after biopsy, having either regressed or been removed entirely by the biopsy. Cervical smears were persistently positive for a year or more in 10 cases, and three of these eventually had invasive carcinoma.

Review of the series of smears from these persistently positive cases showed that the abnormal cell type remained remarkably constant throughout, even over a period of several years. There was one exception, where a sudden progression occurred to a more anaplastic-looking type. In the cases where invasive carcinoma supervened, no alteration in cell type was observed.

The data from this series are consistent with the idea that epithelial instability (basal cell hyperplasia, " carcinoma *in situ* " and related lesions) usually disappears without any treatment more extensive than biopsy, but sometimes becomes irreversible, and in that case sooner or later develops without any cytological warning into invasive carcinoma.

If the smears and biopsies do not become normal within the first year, the lesion should be regarded as precancerous.

We should like to express our thanks to Professor J. Chassar Moir for giving us the facilities of the Nuffield Department of Obstetrics and Gynaecology during a large part of this work ; and to Mr. J. Stallworthy for his constant support and interest. We are very grateful to Dr. A. H. T. Robb-Smith for his advice and help, and to the British Empire Cancer Campaign, from whom A. I. S. and M. M. B. receive whole-time grants. We thank Mrs. J. Shimmin, Mrs. R. Hughes, Miss C. Clarke and Miss G. Olah for their assistance.

### REFERENCES

AYRE, J. E.—(1952*a*) 'Cancer Cytology of the Uterus'. London (Churchill).—(1952*b*) *Sth. med. J., Nashville*, **45**, 915.
BABÈS, A.—(1928) *Pr. méd.*, **36**, 451.
CARSON, R. P. AND GALL, E. A.—(1954) *Amer. J. Path.*, **30**, 15.
FOULDS, L.—(1958) *J. chron. Dis.*, **8**, 2.
GALVIN, G. A., AND TELINDE, R. W.—(1949) *Amer. J. Obstet. Gynec.*, **57**, 15.
*Idem*, JONES, H. W. AND TELINDE, R. W. (1952) *J. Amer. med. Ass.*, **149**, 744.
GRAHAM, R.—(1957) *Acta Cytol.*, **1**, 23.
GUSBERG, S. B. AND MOORE, D. B.—(1953) *Obstet. Gynec.*, **2**, 1.
VON HAAM, E. AND SCARPELLI, D. G.—(1955) *Cancer Res.*, **15**, 449.
HINSELMANN, H.—(1928) *Zbl. Gynäk.*, **52**, 2797.—(1953) *Z. Geburtsh. Gynäk.*, **138**, 153.
HOWARD, L., ERICKSON, C. C. AND STODDARD, L. D.—(1951) *Cancer*, **4**, 1210.
LATOUR, J. P. A., BROWN, L. B. AND TURNBULL, L. A.—(1957) *Amer. J. Obstet. Gynec.*, **74**, 354.
MCCORMACK, L. J., BELOVICH, D. AND KRIEGER, J. S.—(1957) *Amer. J. clin. Path.*, **28**, 179.
MACKENZIE, L. L.—(1955) *Amer. J. Obstet. Gynec.*, **69**, 629.
MARTZLOFF, K. H.—(1923) *Johns Hopk. Hosp. Bull.*, **34**, 141 and 184.
NESBITT, R. E. L.—(1955) *Obstet Gynec.*, **6**, 239.
NIEBURGS, H. E. AND PUND, E. R.—(1950) *J. Amer. med. Ass.*, **142**, 221.

PAPANICOLAOU, G. N.—(1928) *Proc. 3rd Race Betterment Conf.*, 528.—(1954) ' Atlas of Exfoliative Cytology '. Cambridge, Mass. (Commonwealth Fund.)

PETERSEN, O.—(1955) ' Precancerous Changes of the Cervical Epithelium '. Copenhagen (Danish Science Press).

REAGAN, J. W., HAMONIC, M. J. AND WENTZ, W. B.—(1957) *Lab. Invest.*, **6**, 241.

*Idem* AND WENTZ, W. B.—(1959) *A.M.A. Arch. Path.*, **67**, 287.

SCAPIER, J., DAY, E. AND DURFEE, G. R.—(1952) *Cancer*, **5**, 315.

VINCENT MEMORIAL LABORATORY, STAFF OF—(1950) ' The Cytologic Diagnosis of Cancer '. Philadelphia (Saunders).

WIED, G. L.—(1956) *Amer. J. Obstet. Gynec.*, **71**, 793.

YOUNGE, P. A., HERTIG, A. T. AND ARMSTRONG, D.—(1949) *Ibid.*, **58**, 867.

ZINSER, H. K.—(1957) ' Die Zytodiagnostik in der Gynäkologie '. Jena (Fischer).


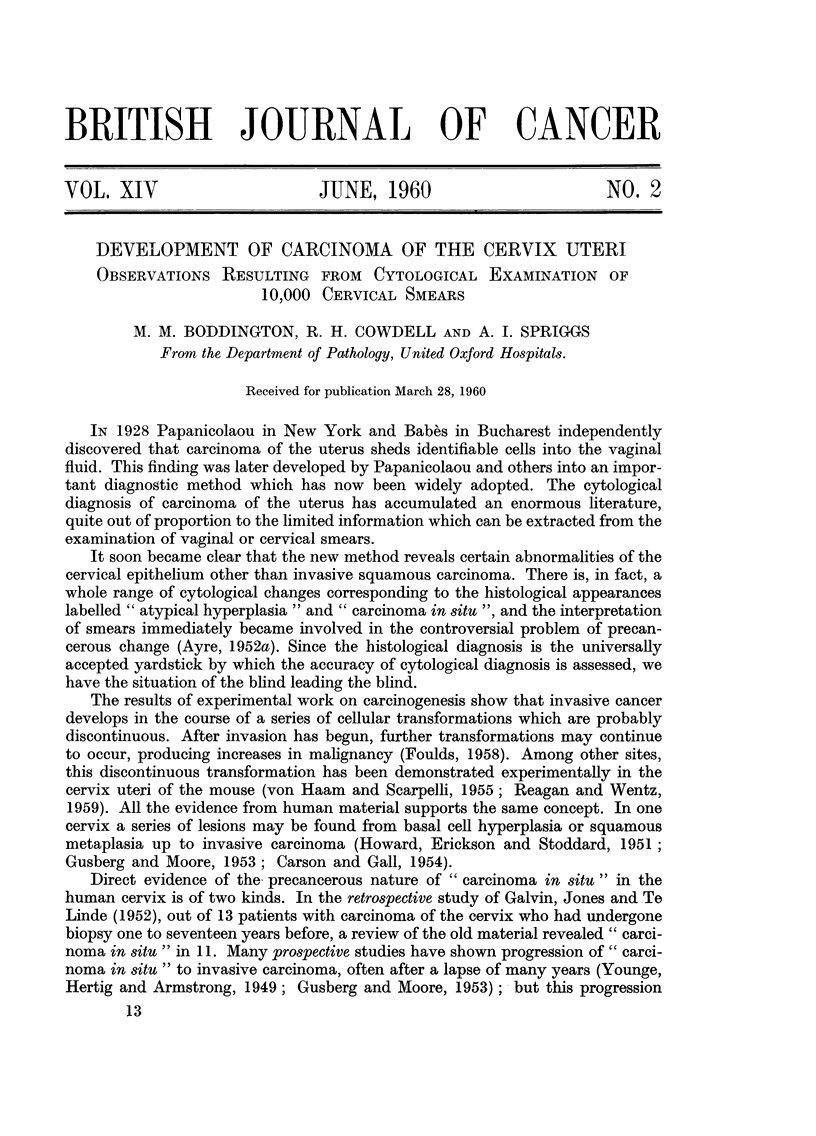

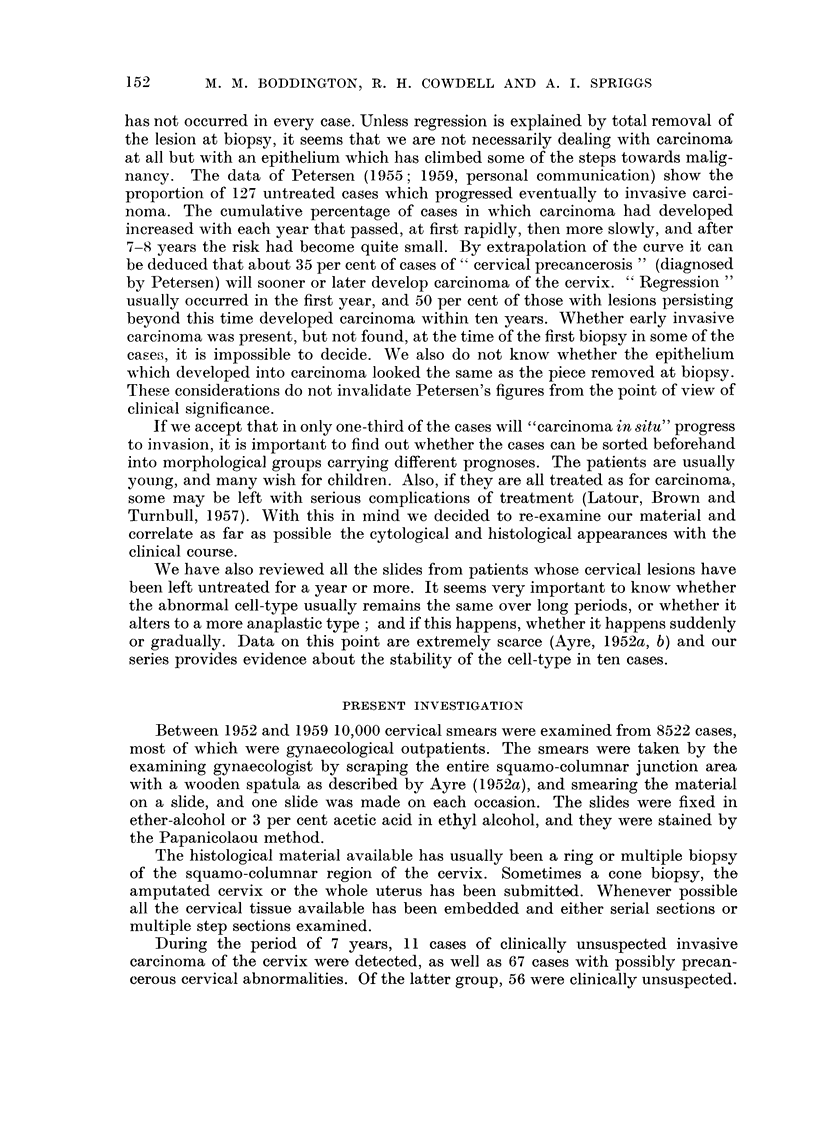

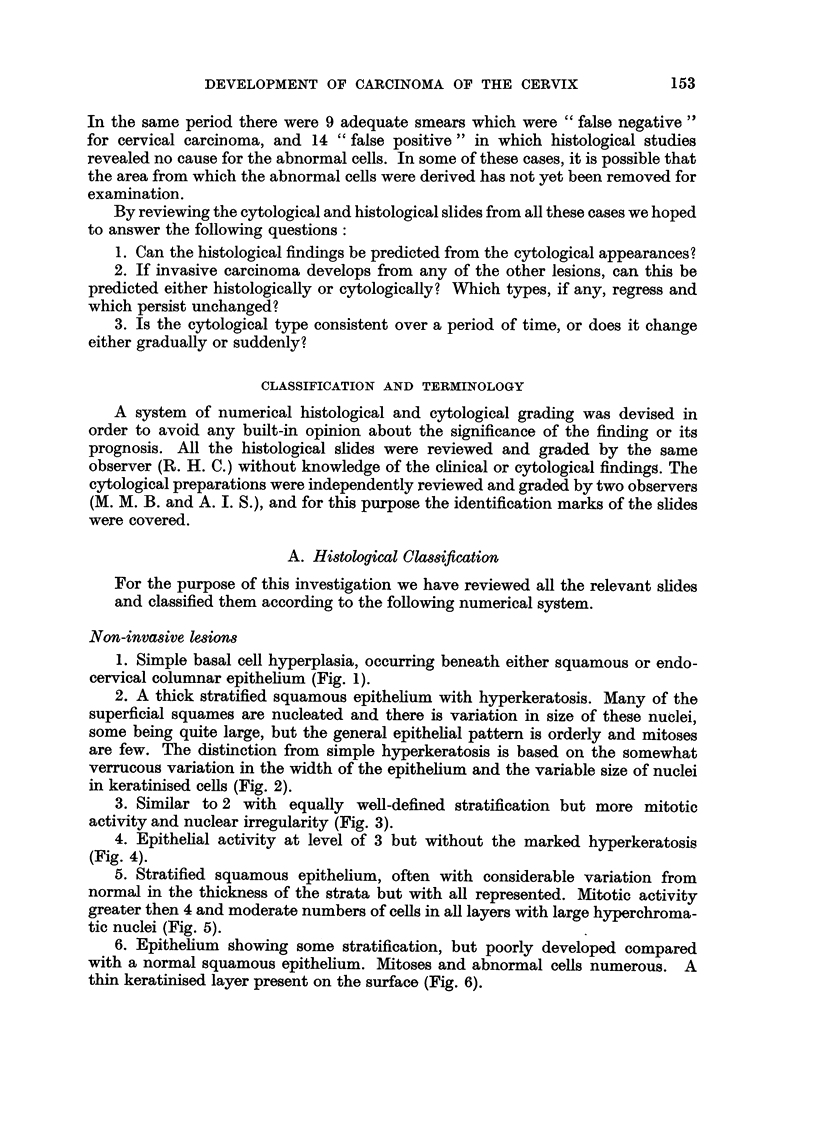

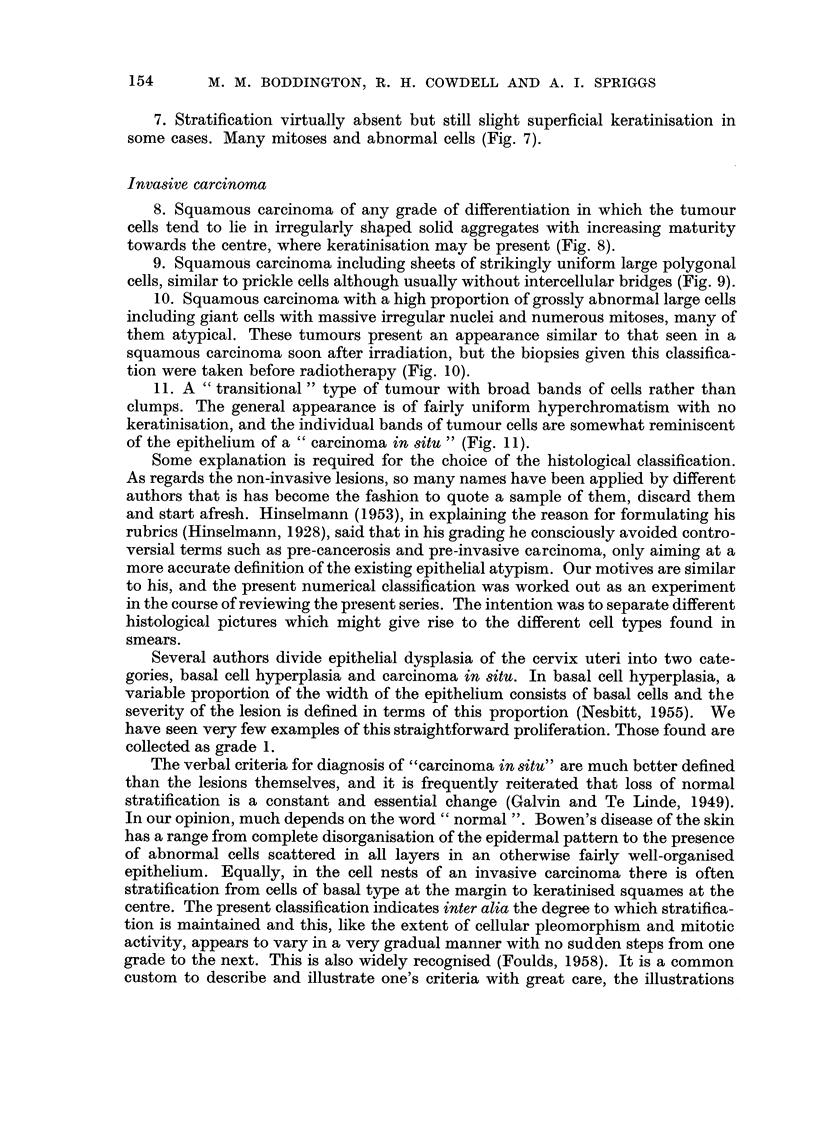

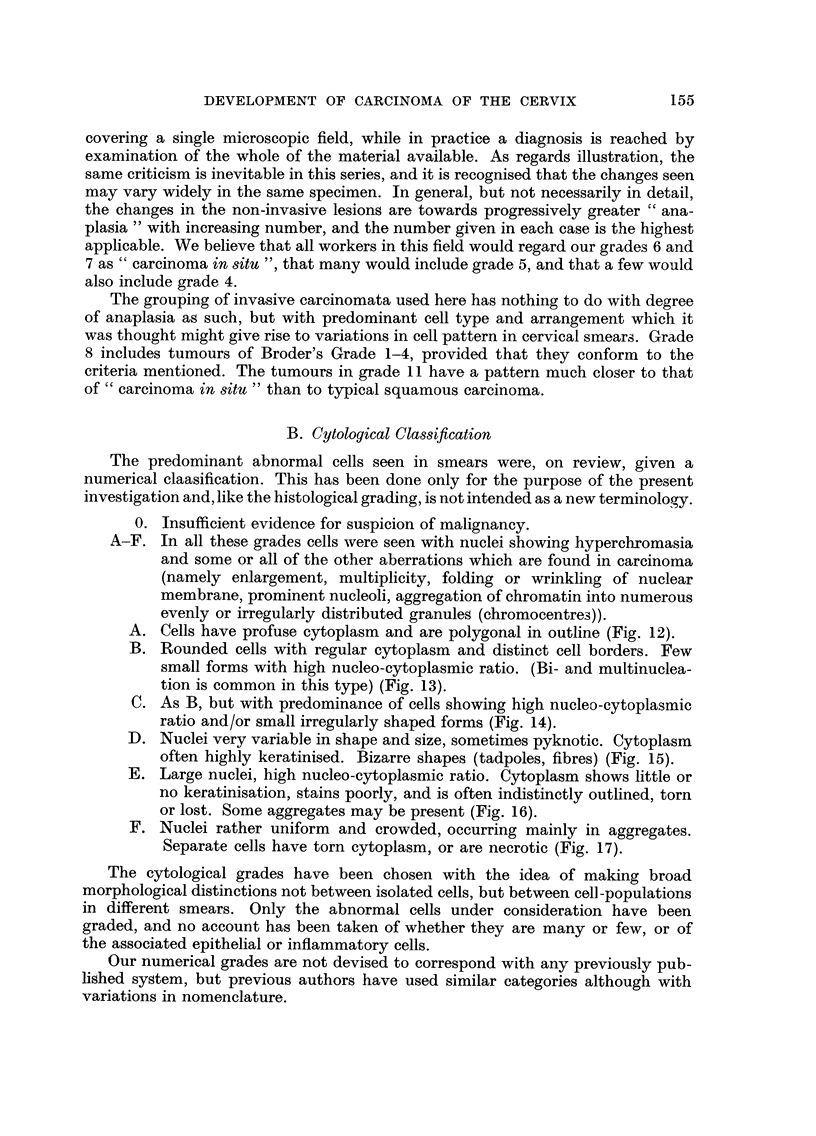

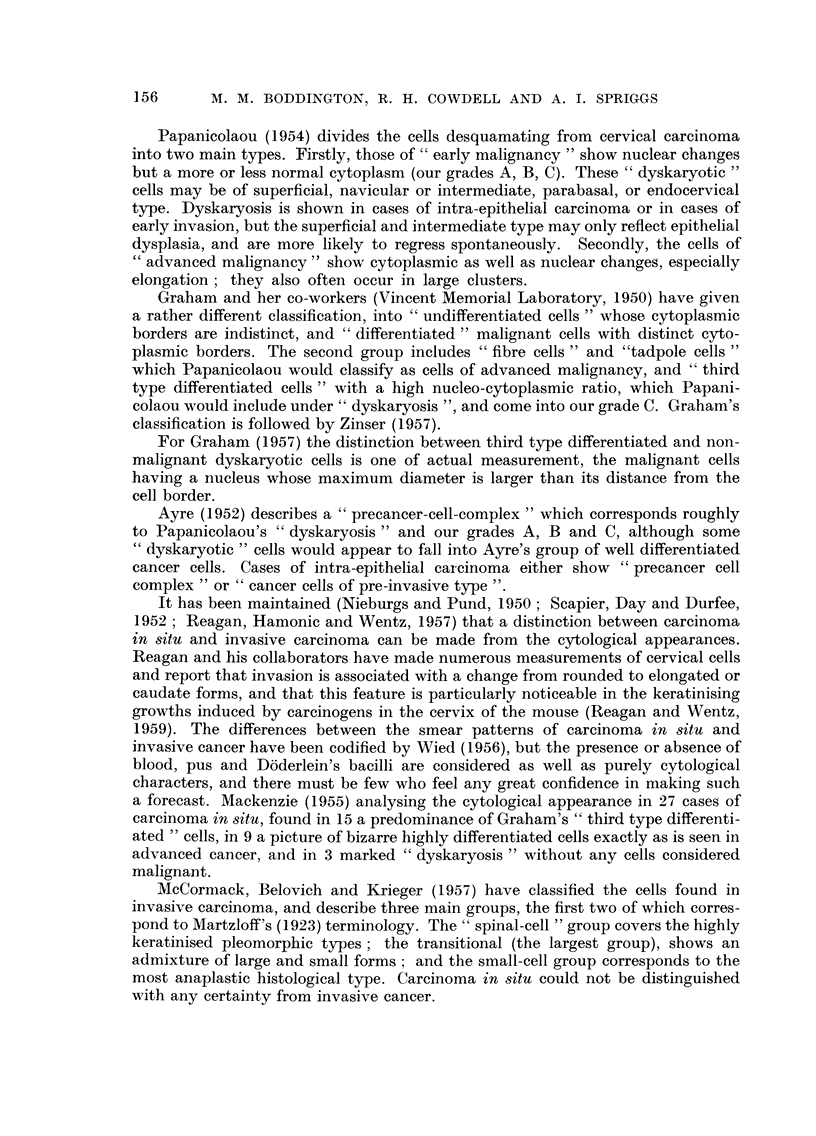

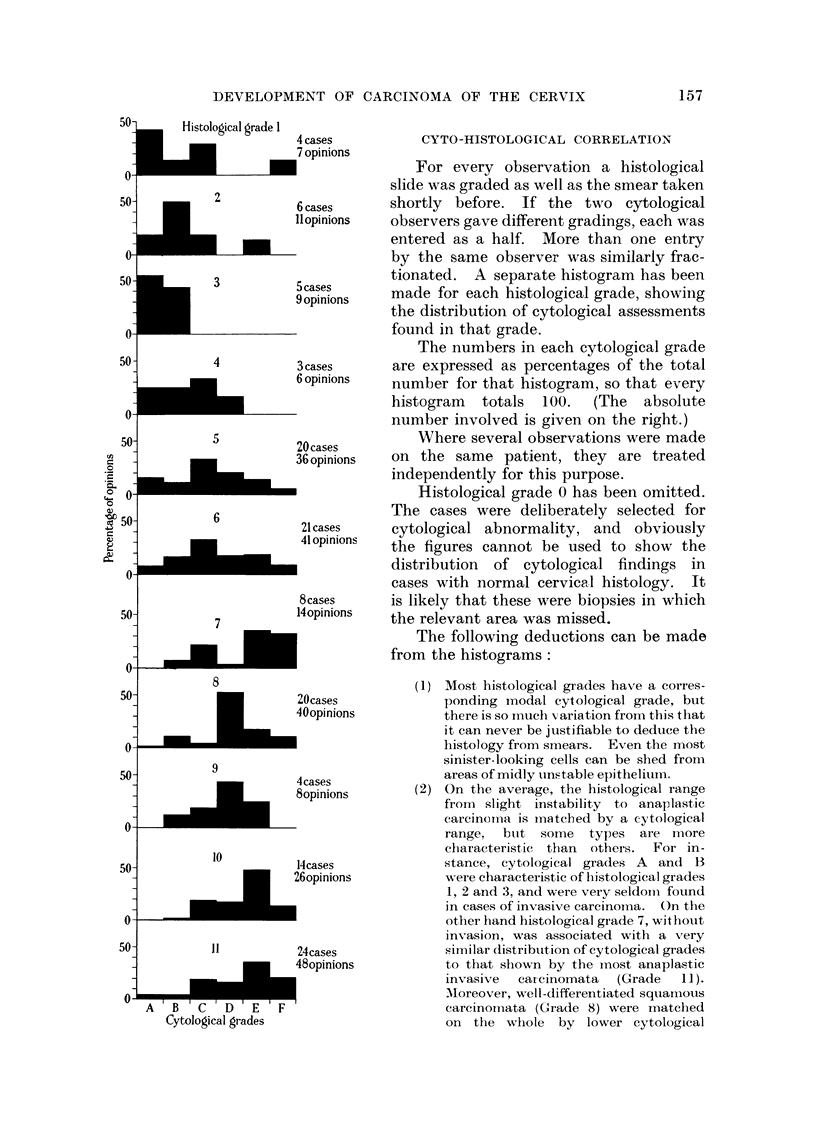

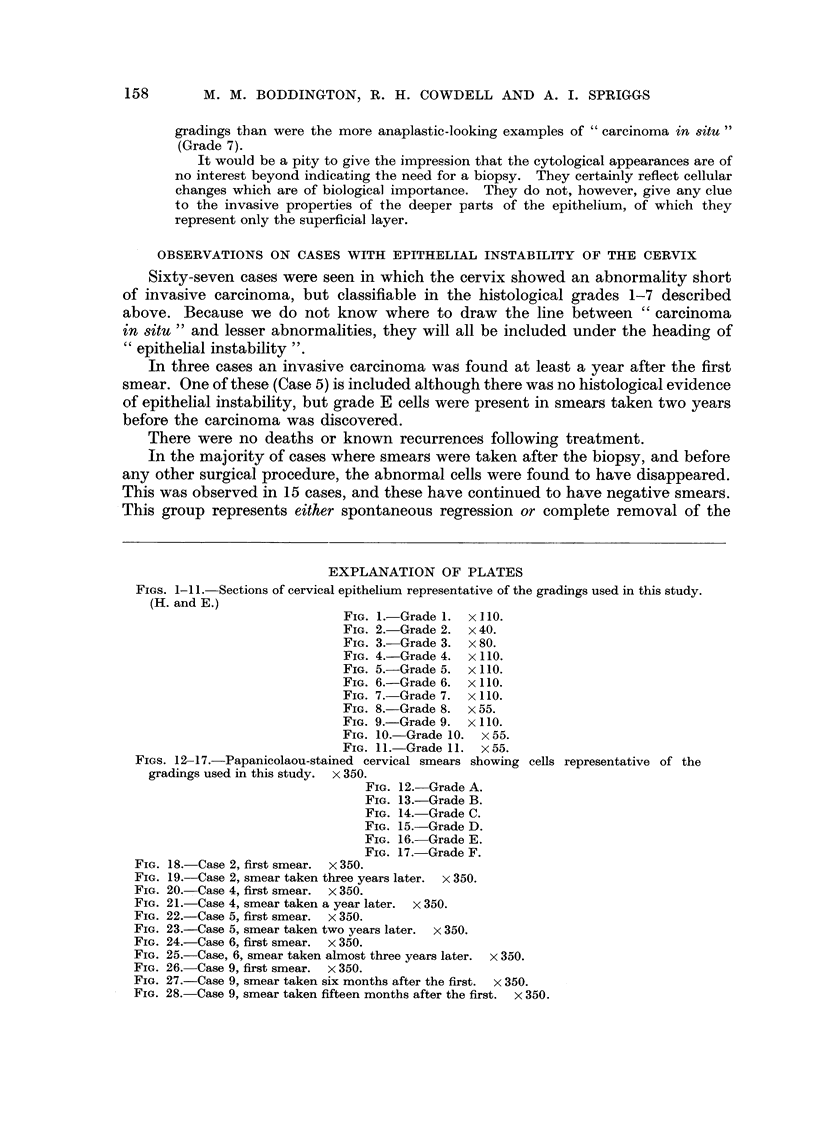

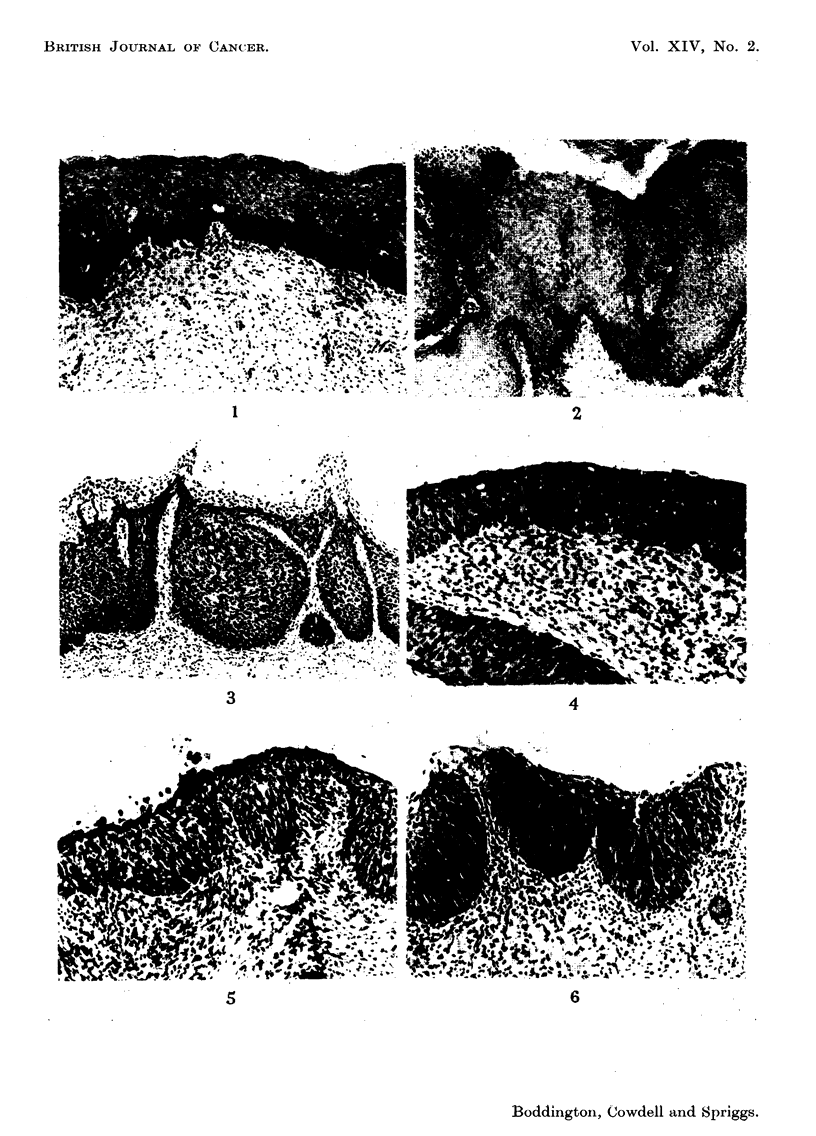

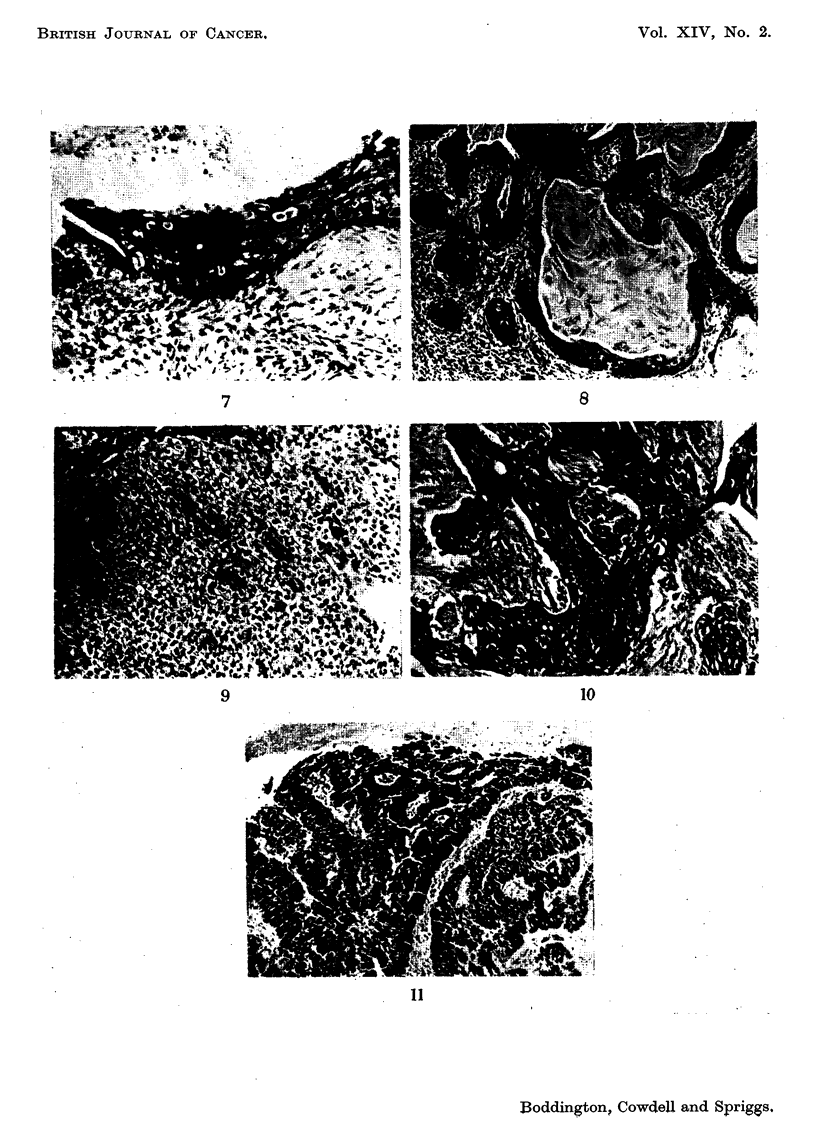

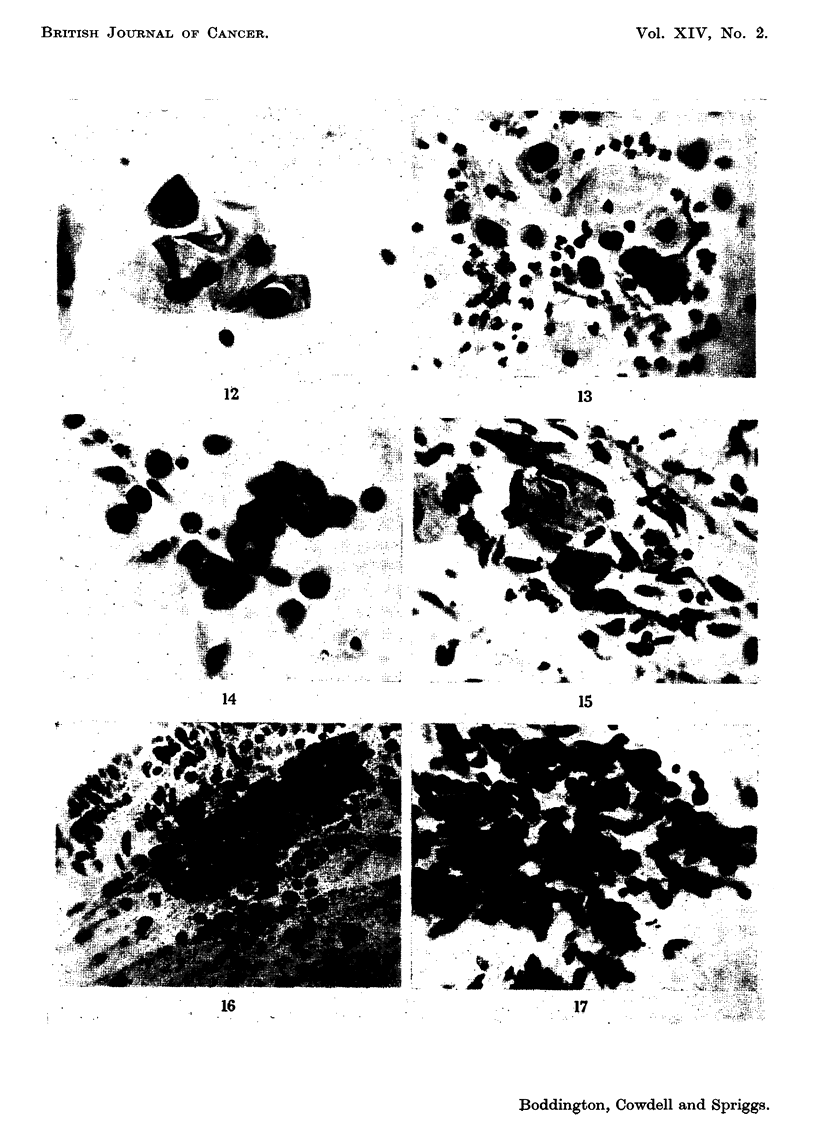

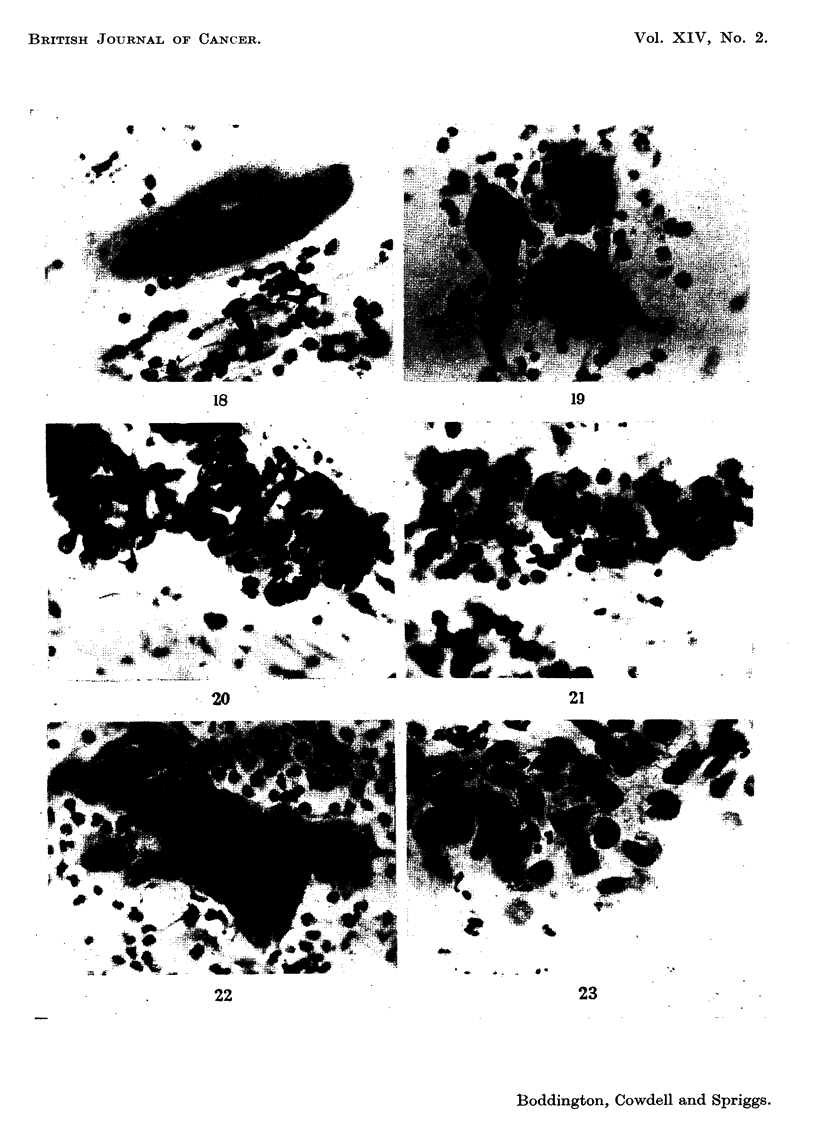

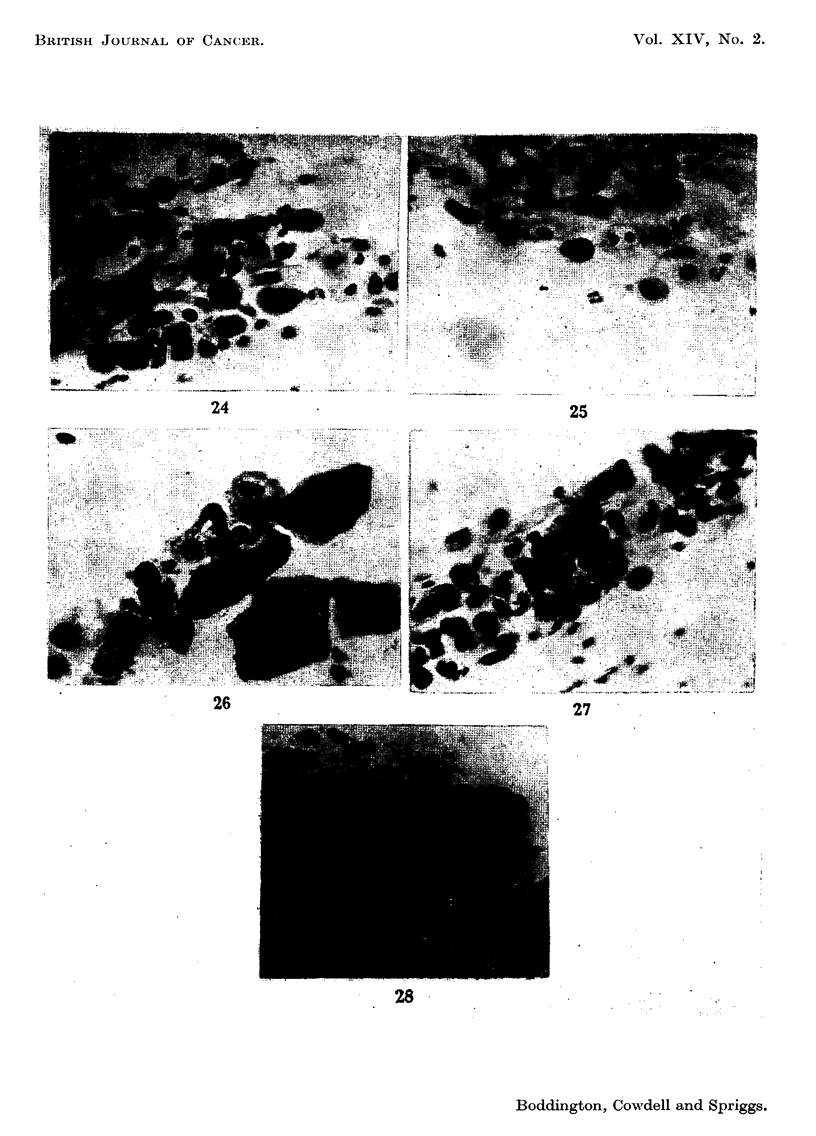

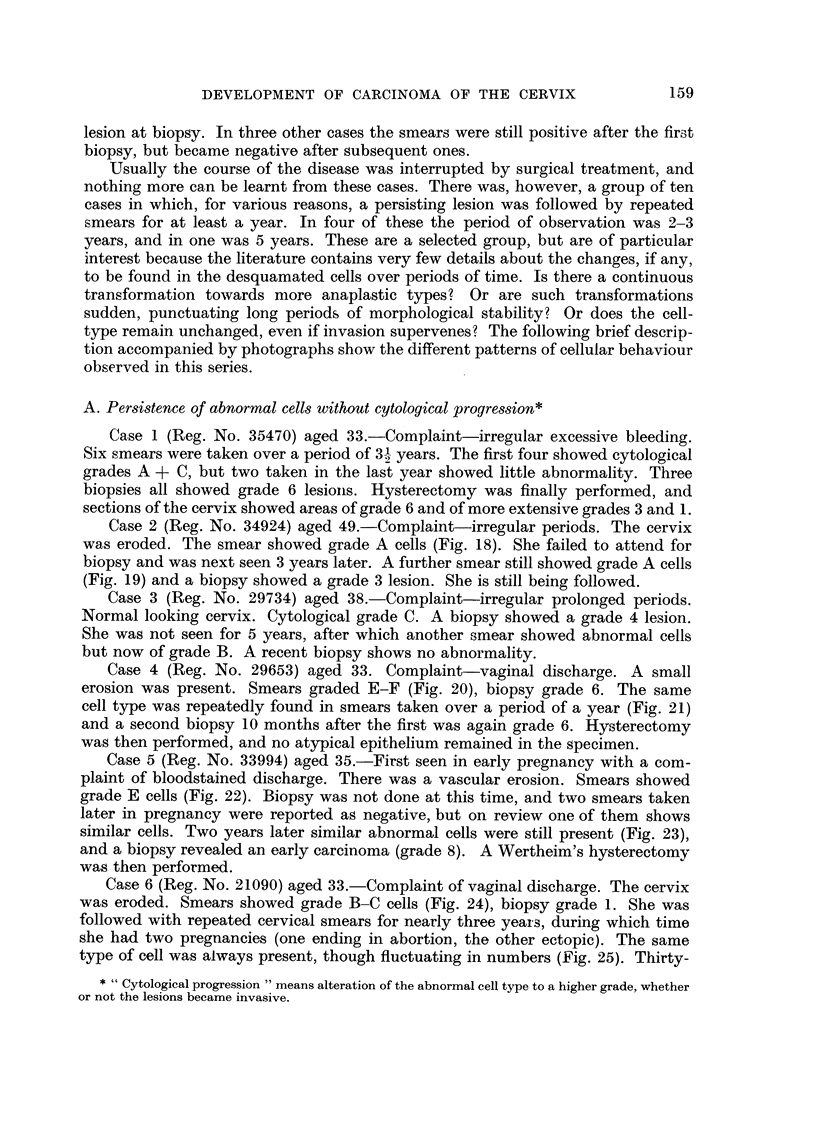

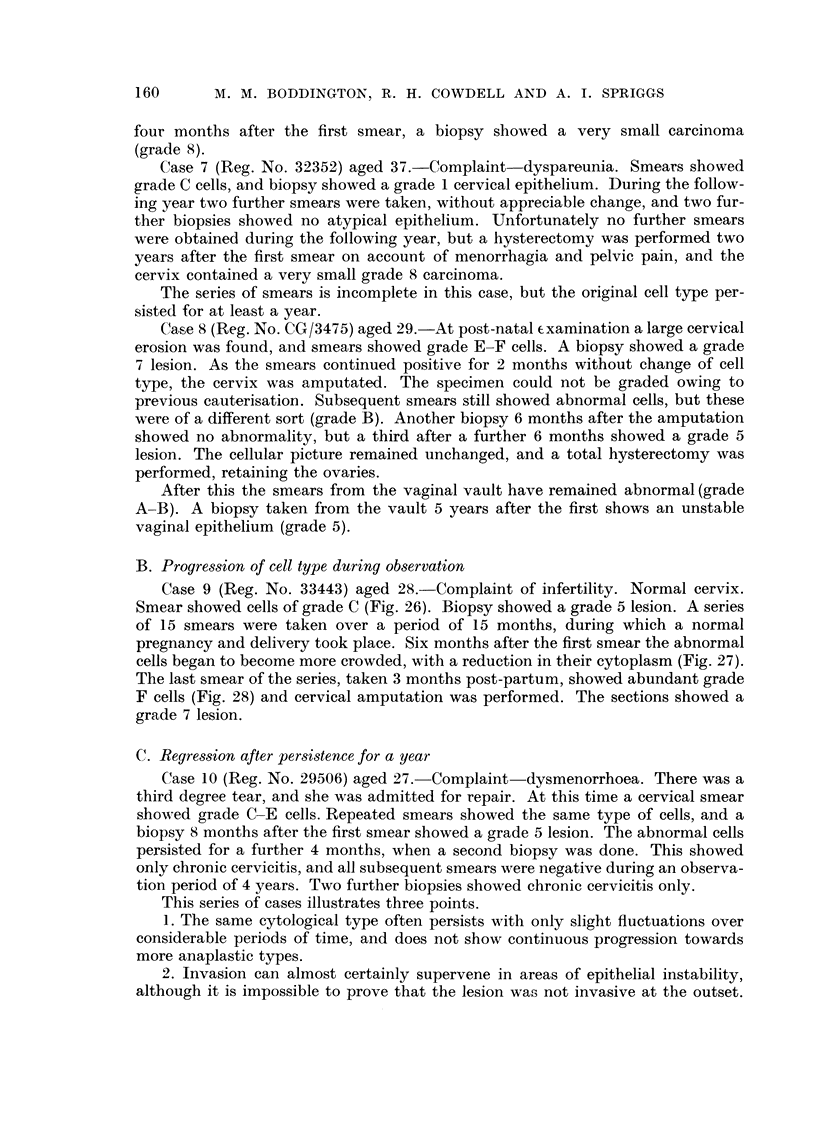

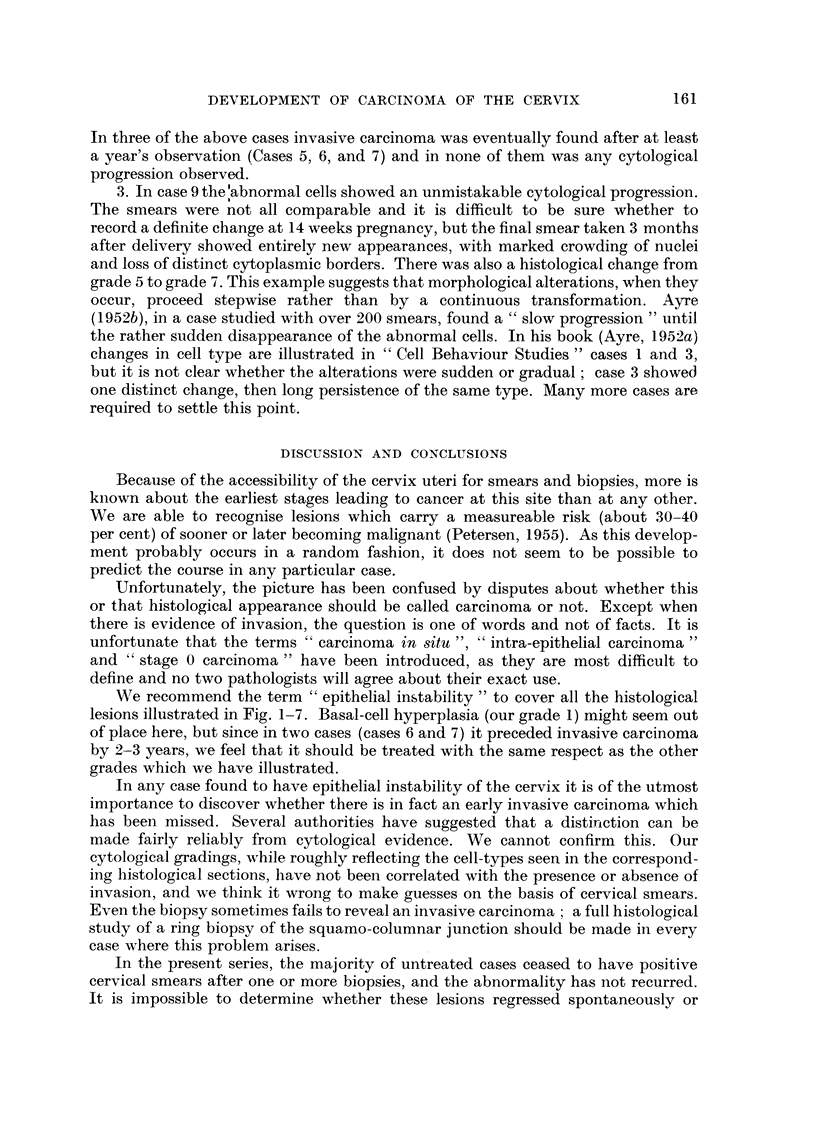

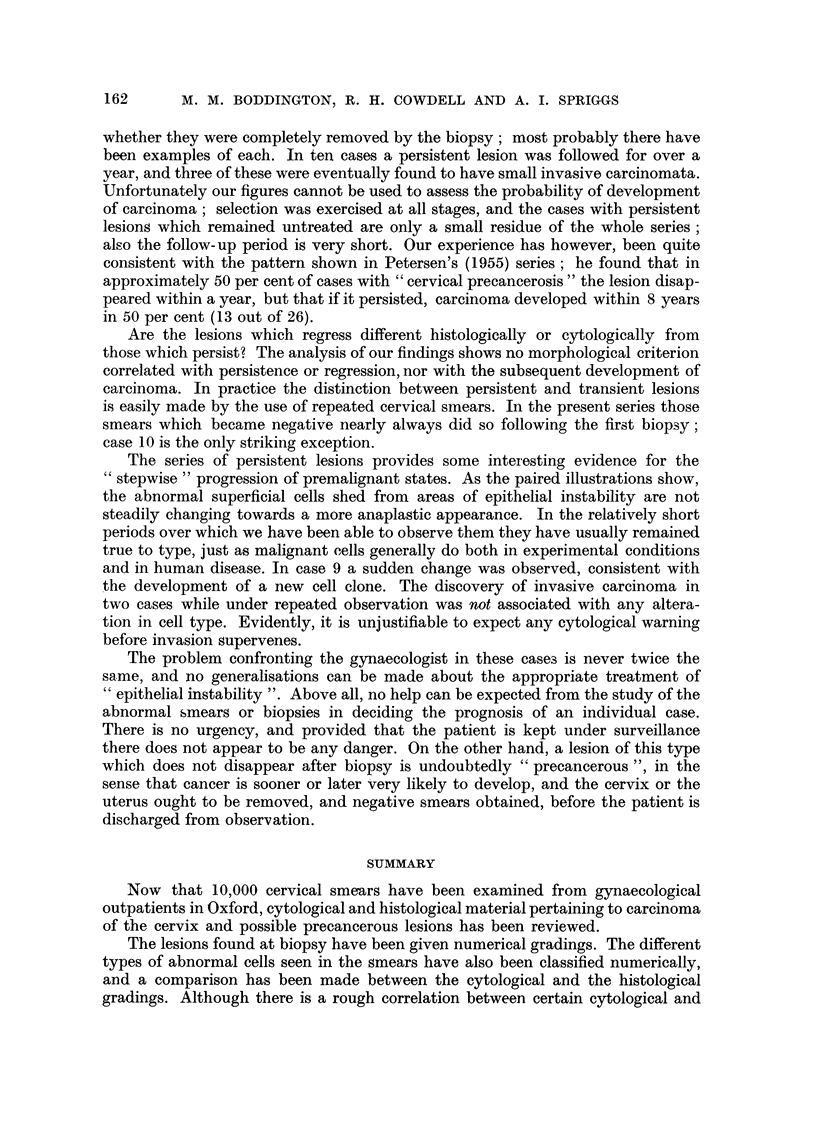

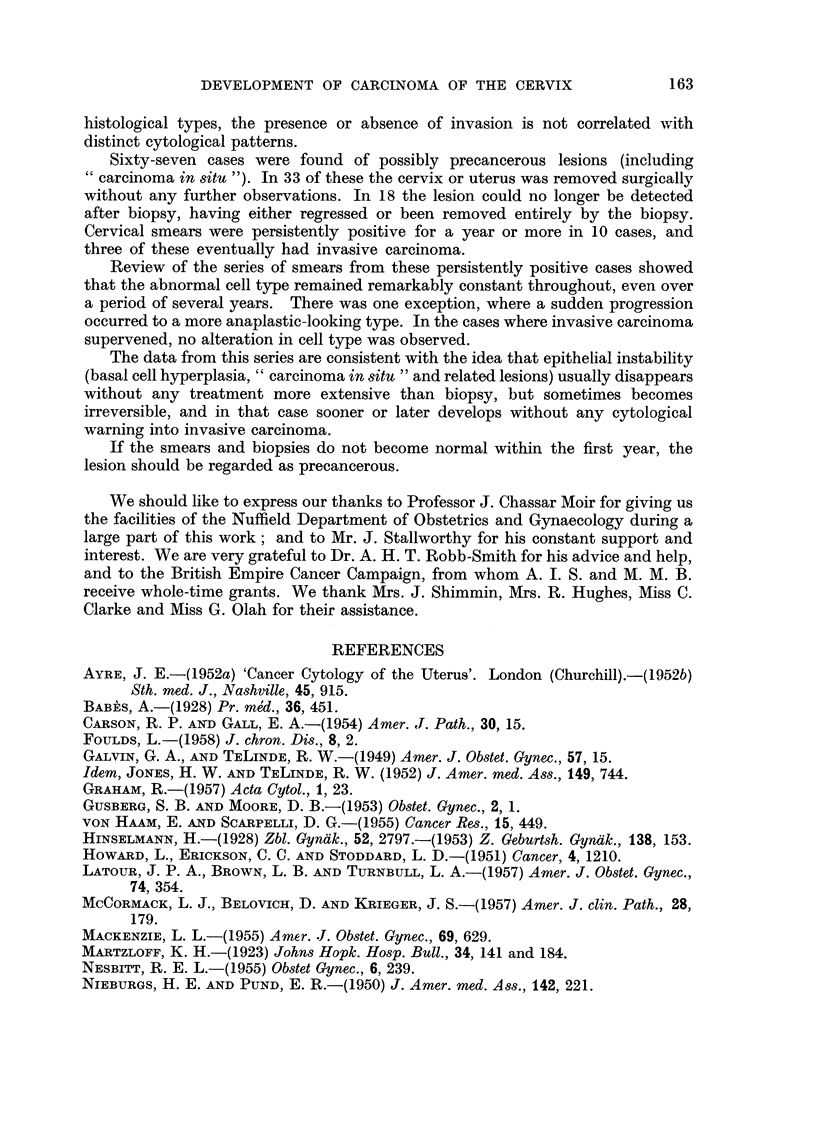

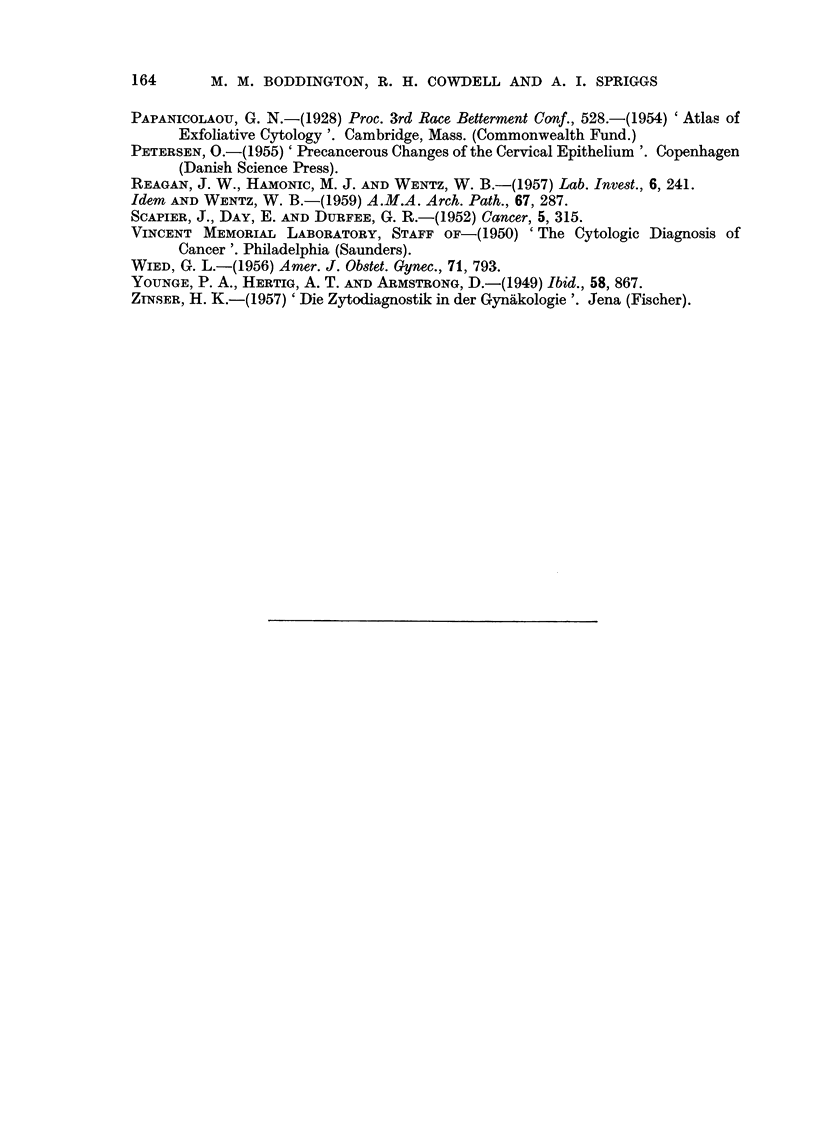


## References

[OCR_01028] GALVIN G. A., JONES H. W., TeLINDE R. W. (1952). Clinical relationship of carcinoma in situ and invasive carcinoma of the cervix.. J Am Med Assoc.

[OCR_01037] HINSELMANN H. (1953). Gesteigert atypisches Epithel oder Oberflächenkarzinom; Entgegnung auf die vorstehende Arbeit von Lax.. Z Geburtshilfe Gynakol.

[OCR_01042] LATOUR J. P., BROWN L. B., TURNBULL L. A. (1957). Preclinical carcinoma of the cervix.. Am J Obstet Gynecol.

[OCR_01050] MACKENZIE L. L. (1955). The cytology of early squamous-cell carcinoma of the cervix.. Am J Obstet Gynecol.

[OCR_01053] NESBITT R. E. (1955). Basal-cell hyperactivity of the cervix in pregnancy with postpartum follow-up.. Obstet Gynecol.

[OCR_01065] REAGAN J. W., HAMONIC M. J., WENTZ W. B. (1957). Analytical study of the cells in cervical squamous-cell cancer.. Lab Invest.

[OCR_01066] REAGAN J. W., WENTZ W. B. (1959). Changes in desquamated cells in carcinogenesis.. AMA Arch Pathol.

[OCR_01068] SCAPIER J., DAY E., DURFEE G. R. (1952). Intraepithelial carcinoma of the cervix; a cytohistological and clinical study.. Cancer.

[OCR_01074] WIED G. L. (1956). The potentialities of the smear technique for the differentiation of noninvasive and invasive cervical carcinoma.. Am J Obstet Gynecol.

[OCR_01076] YOUNGE P. A., HERTIG A. T., ARMSTRONG D. (1949). A study of 135 cases of carcinoma in situ of the cervix at the Free Hospital for Women.. Am J Obstet Gynecol.

